# Maternal capital predicts investment in infant growth and development through lactation

**DOI:** 10.3389/fnut.2023.1272938

**Published:** 2023-10-11

**Authors:** Sarah Dib, Mary Fewtrell, Jonathan C. K. Wells

**Affiliations:** Population, Policy and Practice, UCL Great Ormond Street Institute of Child Health, London, United Kingdom

**Keywords:** maternal capital, life history theory, maternal stress, lactation, infant growth, maternal investment

## Abstract

**Introduction:**

Maternal capital (MC) is a broad term from evolutionary biology, referring to any aspects of maternal phenotype that represent resources available for investment in offspring. We investigated MC in breastfeeding mothers of late preterm and early term infants, examining its relationship with infant and breastfeeding outcomes. We also determined whether MC modified the effect of the relaxation intervention on these outcomes.

**Methods:**

The data was collected as part of a randomized controlled trial investigating breastfeeding relaxation in 72 mothers of late preterm and early term infants. Indicators of MC (socioeconomic, social, somatic, reproductive, psychological, and cognitive) were collected at baseline at 2–3 weeks post-delivery. Principal Component Analysis was conducted for the MC measures and two components were identified: 1.”Subjective” maternal capital which included stress and depression scores, and 2.”Objective” maternal capital which included height, infant birth weight, and verbal memory. Univariate linear regression was conducted to assess the relationship between objective and subjective MC (predictors) and infant growth, infant behavior, maternal behavior, and infant feeding variables (outcomes) at 6–8 weeks. The interaction of MC and intervention assignment with outcomes was assessed.

**Results:**

Higher objective MC was significantly associated with higher infant weight (0.43; 95%CI 0.21,0.66) and length z-scores (0.47; 95%CI 0.19,0.76), shorter duration of crying (−17.5; 95%CI −33.2,−1.9), and lower food (−0.28; 95%CI −0.48,−0.08) and satiety responsiveness (−0.17; 95%CI −0.31,−0.02) at 6–8 weeks. It was also associated with greater maternal responsiveness to infant cues (−0.05, 95%CI −0.09,−0.02 for non-responsiveness). Greater subjective maternal capital was significantly associated with higher breastfeeding frequency (2.3; 95%CI 0.8,3.8) and infant appetite (0.30; 95%CI 0.07,0.54). There was a significant interaction between the intervention assignment and objective MC for infant length, with trends for infant weight and crying, which indicated that the intervention had greater effects among mothers with lower capital.

**Conclusion:**

Higher MC was associated with better infant growth and shorter crying duration. This was possibly mediated through more frequent breastfeeding and prompt responsiveness to infant cues, reflecting greater maternal investment. The findings also suggest that a relaxation intervention was most effective among those with low MC, suggesting some reduction in social inequalities in health.

## 1. Introduction

Lactation is an ancient reproductive feature with a long evolutionary history that is thought to predate the origin of mammals, more than 200 million years ago (1, 2). In modern mammals, the primary role of breast milk is to provide a complete source of nutrition. However, it also provides a medium through which ‘signaling’ or communication between the mother and offspring can occur. From an evolutionary perspective such signaling is expected on the grounds that the magnitude of nutritional investment that maximizes maternal Darwinian fitness will not be identical to that which maximizes the Darwinian fitness of each offspring, as the two parties share only 50% of their alleles (3). Signaling through lactation might occur by altering milk production, milk energy transfer, milk composition, and/or the duration of lactation; all of which are aspects of lactation strategy and vary between and within species. Some examples of these lactation strategies include arctic hooded seals feeding for a short period milk of very high energy and fat concentration in response to unstable and cold climates, or bats delivering small volumes of concentrated milk to avoid impairing the ability to fly (4, 5). Therefore, signaling enables the mother to alter investment in the offspring and regulate their growth and behavior according to her phenotype and to changing environmental circumstances (6).

There is compelling evidence that the magnitude of maternal investment, including lactation, has long-term implications for the health and Darwinian fitness of the offspring (7–12). The classic ‘thrifty phenotype’ hypothesis of Hales and Barker (13) proposed that low levels of maternal investment, as indicated by low birth weight, increase the risk of cardiometabolic risk and diabetes in adulthood, especially if overweight develops. The thrifty phenotype hypothesis prompts the question, why should variability in maternal investment have such long-term effects on the health of the offspring?

One evolutionary perspective on this developmental association was the predictive adaptive response (PAR) hypothesis, which assumes that maternal investment in early life provides a signal of the likely ecological conditions that will be encountered by the offspring in adulthood, which therefore calibrates its phenotype to this signal (14). If the adult environment turns out to be different (‘mismatch’), then the risk of non-communicable disease is assumed to increase. However, the PAR hypothesis has been criticized on both empirical and theoretical grounds. Empirical tests fail to show that survival and fitness in adult environments of famine are improved among those who experienced famine in early life (15). From a theoretical perspective, the notion that environments will remain constant over long time periods has also been questioned (16, 17).

An alternative evolutionary perspective is that the signals provided through maternal investment in early life, including those transferred through breast milk, do not reveal information directly about the external environment, but rather about maternal phenotype itself (17). Within any given setting, mothers may vary in their capacity to invest in offspring, examples being variability in maternal energy stores, parity and socioeconomic status. Some maternal traits may be determined in the mother’s own early life (e.g., height and resting metabolic rate), while others may vary on shorter timescales (body fat stores, psychological state) (17). Even when a stimulus emerges from the environment (e.g., stress), maternal phenotype (the stress response) may still mediate the exposure that is actually experienced by the offspring.

Building on the embodied capital approach of Kaplan and colleagues (18), aspects of maternal phenotype that enable differential investment in the offspring have been defined as “maternal capital” (17). Maternal capital is an umbrella term that encompasses several broad categories of traits, including somatic capital (e.g., body weight, height and composition), social capital (e.g., support networks), cognitive capital (e.g., knowledge and skills acquired from formal education, or informally), psychological capital (e.g., resilience to psychological distress) and material capital (e.g., financial income and savings, housing quality). Overall, maternal capital represents the environment to which the offspring is directly exposed in early-life (17), and thus different levels of maternal capital may produce varying levels of investment in the offspring, thereby shaping offspring phenotype and development.

**Figure 1:**
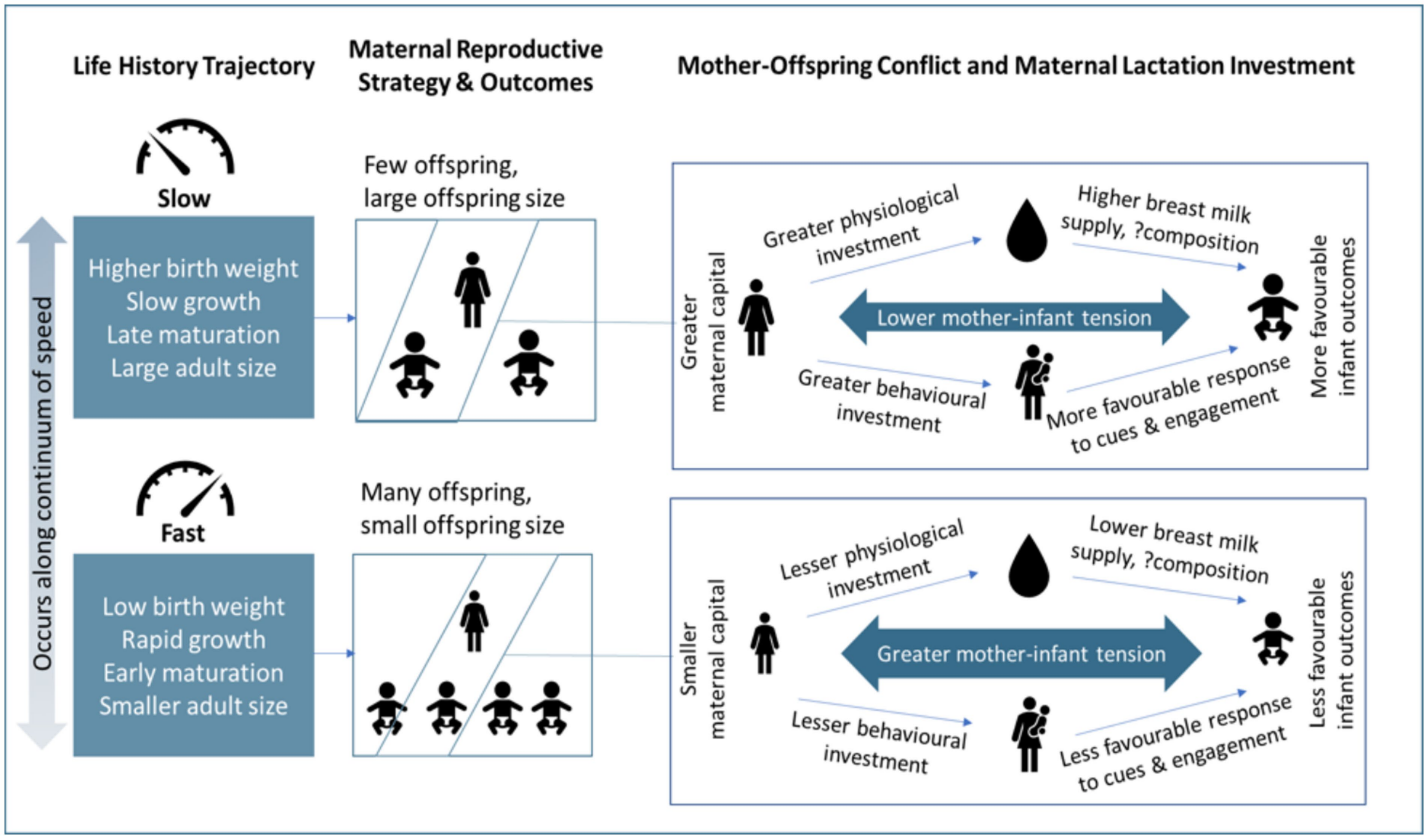
The relationship between life history theory, maternal reproductive strategy, mother-offspring conflict and maternal capital on lactation investment and infant outcomes. The figure shows that slower life history trajectories often favor reproducing fewer offspring and investing more in each offspring. In a more detailed perspective, slower life history trajectories often translate to greater maternal capital and thus lower mother-infant tension over resources and greater investment in the offspring through physiological (breast milk) and/or behavioral pathways.

The magnitude of maternal capital is influenced by maternal life-history trajectory (19). Life-history theory is based on the principle of thermodynamics, and holds that energy used for one purpose cannot be used for another (20). Organisms harvest energy from their environment, and invest this energy in competing biological functions, resulting in trade-offs between them. The four main functions competing for energy are maintenance (including repair of cells and tissues), growth, immunity or defense against predators, and reproduction (20, 21). The strategies by which energy is allocated can be described along a continuum of the pace of the life-course (22). ‘Slow’ life history patterns are favored when mortality risk is low, and are characterized by slower growth, delayed maturation, producing fewer offspring but investing more in each, and reduced risk taking. Conversely, ‘fast’ patterns are favored when mortality risk is high, and are characterized by early maturation, rapid growth, producing many offspring but investing little in each, and more risky behaviors. In high-risk situations, organisms generally favor rapid growth and reproduction over maintenance and defense in order to maximize the chances of reproducing before mortality occurs (21). Maternal life-history trajectories can further influence those of their offspring, indicating intergenerational dynamics. For example, many studies have demonstrated that age at menarche in mothers is strongly associated with age at menarche in daughters (23–26), and with timing of pubertal development in sons and daughters (23, 27). Additionally, earlier age at menarche for mothers has been shown to be associated with higher offspring BMI (28, 29) and more rapid weight gain in infancy (29), suggesting that a ‘faster’ maternal life-history trajectory might predict a ‘faster’ offspring trajectory. Overall, we can expect that mothers with slower life-history trajectories would produce fewer, larger offspring and have greater capital available for investment in each (Figure 1).

Studies have consistently shown that individual aspects of maternal capital are associated with infant and childhood outcomes. For example, data from India, Peru and Vietnam showed that maternal social capital, specifically group membership, was associated with higher infant birth weight across the three countries (30). Humans are cooperative breeders and group members, or alloparents, can provide protection, care and provisioning to the offspring (31). This in turn lowers the direct costs of parental care to the mother, and allows her to invest more in her own maintenance and her future reproduction. This is especially evident in many traditional societies that practice a confinement/rest/quarantine period postpartum, where the mother is provided with help with household chores, her health, infant care and breastfeeding/infant feeding by family members (32–34). Other studies have shown that maternal anthropometry (somatic capital) was positively associated with infant birth weight (35, 36) and childhood educational attainment (37). Psychological capital is also important to consider. In wild olive baboons, mothers who experienced more early life adversity had higher concentrations of glucocorticoids, and subsequently spent more time nursing their offspring (as a possible reflection of more dilute or lower volumes of milk produced) (38). This study also found that high social status buffered against some forms of early life adversity, where some aspects of early life adversity had larger effects on mortality, nursing, and carrying among lower ranking mothers.

In some cases, it may be helpful to evaluate maternal capital as a composite trait, recognizing that mothers with better social circumstances may also have more favorable somatic capital. For instance, an analysis of a birth cohort in Brazil combined data on maternal height, pre-pregnancy BMI, income and education to create a score reflecting overall maternal capital (39). The results indicated that low maternal capital was associated with clustering of adverse outcomes in the daughters, such as lower birth weight, early reproduction, school dropout, shorter adult height and more central fat distribution. It is already acknowledged in biomedical research that many aspects of maternal characteristics are associated with infant growth and development. However, it may also be informative to evaluate different aspects of maternal capital simultaneously, and to examine these relationships from an additional anthropological lens.

From a public health perspective, strategies that enhance maternal capital could potentially improve children’s health and development by improving the environment the offspring is exposed to during critical windows of growth (40). Ideally, interventions should target women pre-conception, as maternal capital is a reflection of the mother’s own development and past and current experience. However, strategies during pregnancy and lactation are also essential, especially for high-risk women to mitigate the effects of capital penalties.

Breast-feeding is clearly a key component of maternal investment, where the mother supplies not only the nutrients required for metabolism and growth, but also primes the offspring’s immune system and thus supports ‘defense’. Reflecting the discussion above, the exact nature of this investment is expected to vary in association with components of maternal capital. To date, most attention on this association has focused on somatic traits, such as maternal BMI (41), or socio-economic traits such as household wealth and maternal education (42–44). However, there is growing evidence that maternal psychological state is associated with the volume and composition of breastmilk that the infant consumes (45–48). We previously showed in a randomized controlled trial that reducing psychosocial stress, by asking breastfeeding mothers of late preterm and early term infants to listen to a breastfeeding meditation audio, resulted in higher infant weight gain (49). Not only did the intervention yield greater investment in the offspring but also in the mother herself, as evidenced by better verbal memory scores of mothers in the intervention group.

This paper investigates maternal capital in the same sample of breastfeeding mothers of late preterm and early term infants, and examines its relationship with infant, maternal, and breastfeeding outcomes. It also determines whether the relaxation intervention is able to modify or moderate the relationship between maternal capital and infant, maternal and breastfeeding outcomes.

## 2. Methods

### 2.1. Study design

The data was collected as part of a randomized controlled trial investigating a breastfeeding relaxation in mothers of late preterm and early term infants in London, United Kingdom. The main trial results were published elsewhere (50). Briefly, mothers of healthy infants of 34^0/7^ to 38^6/7^ gestational weeks were identified and screened before discharge from three hospitals in London. Mothers were eligible if they had a singleton pregnancy, intended to breastfeed for at least 6 weeks, spoke and understood English, did not smoke, were free of serious illness, and did not have a prior breast surgery that could interfere with breastfeeding. Seventy-two participants provided informed consent and were randomized to the relaxation group, where the audio was provided, or to the control group where no intervention was given. The study was ethically approved by the Health and Research Authority in the United Kingdom (IRAS:252031) and registered with ClinicalTrials.gov (NCT03791749).

### 2.2. Data collection

#### 2.2.1. Maternal capital (predictors)

**Figure 2:**
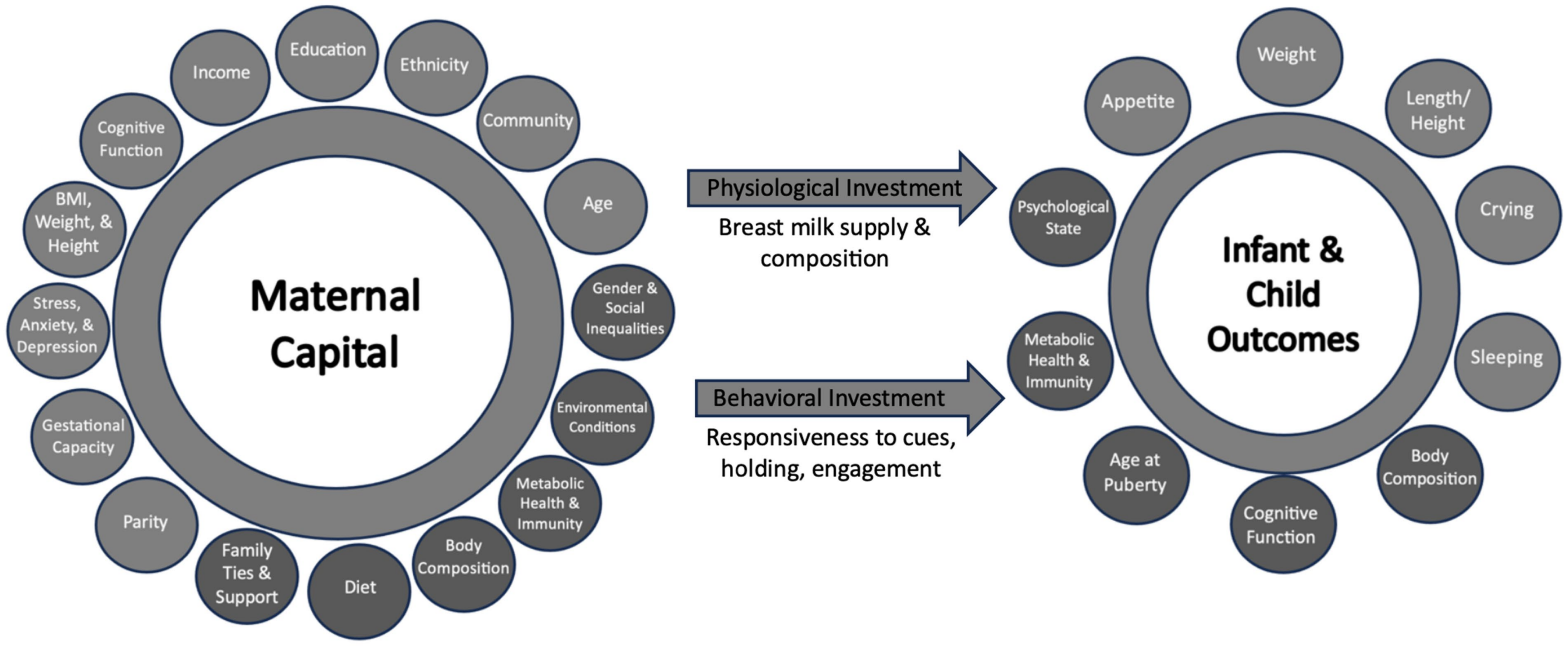
Proposed indicators of maternal capital, maternal investment and infant outcomes. Light circles represent the variable collected and considered in the principal component analysis (PCA), while dark circles represent variables not collected in this study.

Data that was deemed indicative of maternal capital, i.e., reflective of maternal capital budget was included for this analysis (Figure 2). Detailed description of data collection is mentioned elsewhere (49, 50). In summary, indicators of maternal capital (socioeconomic, social, somatic, reproductive, psychological, and cognitive) were collected at 2–3 weeks post-delivery, prior to providing the relaxation intervention for those in the intervention group. Participants were asked to report their income, education level, amount of weight gained during pregnancy, height, parity and the infant’s birth weight (as an indication of nutritional investment during pregnancy). When measuring the participant’s weight at 2–3 weeks was not feasible, mothers were asked to report their current weight, if known. Maternal psychological state at baseline was assessed using the Perceived Stress Scale (51) and Edinburgh Postnatal Depression Scale (52), for stress and depression, respectively. Maternal investment in helping others in the community was assessed at baseline using the Helping Attitude Scale, which is a 20-item measure of the participant’s beliefs, feelings, and behaviors associated with helping (53). Each item is answered on a 5-point Likert scale, ranging from 1 (strongly disagree) to 5 (strongly agree). Higher scores indicate “better” helping attitude.

Verbal memory, is one measure of cognitive function which could be influenced by various short- and long-term factors such as hormones, psychological state, education and skills. Therefore, verbal memory can serve as an indicator of cognitive capital which might differentially influence investment in the offspring. We assessed verbal memory using Rey’s Auditory Verbal Learning Test (RAVLT) (54) at 2–3 weeks. RAVLT consists of 5 recall trials (Trial 1–5) using List A (15 noun word list) and one recall after an interference period (Trial 6) using List B (another 15 noun word list). During each of the first 5 trials, 15 words (from List A) were presented to the participant (with a 1-s interval between each word) after which they were asked to recall as many words as possible. The recall after each trial was recorded. An interference list was then read to the participants consisting of 15 other words (List B) and they were again asked to recall as many nouns as possible. Immediately after, the participants were asked to recall words from List A (Trial 7). The outcomes of the test include the following based on previously described criteria: 1. Immediate recall: sum of correct responses after first 5 trials (Trial 1–5), 2. Verbal learning: difference in number of correct responses after Trial 5 and Trial 1, and 3. Verbal forgetting: difference between correct responses after Trial 7 and Trial 5. Higher immediate recall, higher verbal memory and lower verbal forgetting are indicative of better verbal memory. It is expected that mothers who have higher maternal capital budget would have better verbal memory.

#### 2.2.2. Infant and maternal investment outcomes

Data on infant and breastfeeding variables were collected at 6–8 weeks post-delivery. In summary, infant variables were weight-for-age and length-for-age z-scores, appetite assessed using the Baby Eating Behavior Questionnaire (55) and duration of crying and sleeping assessed using a 3-day baby behavior diary (56). Appetite included four eating traits: ‘enjoyment of food’, ‘food responsiveness’, ‘slowness in eating’ and ‘satiety responsiveness’. Enjoyment of food and food responsiveness indicate a greater appetite or interest in food as perceived by the parent, whereas the other two categories indicate a better appetite control or lower interest in milk.

Outcomes related to maternal investment in breastfeeding were breast milk macronutrient composition, breast milk volume supplied at the breast (estimated using a 24-h breastfeeding diary- method described in (49)), the number of expressed breast milk bottles and formula bottles provided in the previous 7 days, exclusive breastfeeding status, and the frequency of direct breastfeeding in a day. Outcomes related to behavioral investment in the infant were attachment to the infant (using Maternal Attachment Index (57)) and the extent to how prompt the mother responds to infant cues [using Maternal Responsiveness Questionnaire (58)].

### 2.3. Relaxation intervention

As part of the randomized controlled trial described in (49, 50), a breastfeeding meditation audio was offered to breastfeeding mothers of late preterm and early term infants who belonged to the intervention group. Mothers were asked to listen to the 11-min guided-imagery meditation audio that contained breastfeeding messages, at least once a day for at least 2 weeks between 2–3 weeks and 6–8 weeks post-delivery. The main target of the intervention was to reduce mother-infant tension over resources, by reducing maternal stress, and consequently improve infant weight gain.

### 2.4. Statistical analysis

Principal Component Analysis was conducted for the maternal capital measures (mentioned in Section 2.2 and in Table 1) to create composite scores. Kaiser-Meyer-Olkin Measure of Sampling Adequacy and the Bartlett test of sphericity were used to assess whether PCA is appropriate. Eigenvalues (of 1), scree plots, and parallel analysis were used to identify the relevant components of maternal capital. It was found that PCA was appropriate (KMO = 0.563) for a few measures: maternal height, infant birth weight, perceived stress score, depression score, and verbal learning. Two components were identified: 1. “Subjective” maternal capital which included stress and depression scores, and 2. “Objective” maternal capital which included height, infant birth weight, and verbal learning. Regression factors were saved and objective and subjective maternal capital were also each transformed into two categories: low maternal capital which included participants in the bottom third percentile, and high capital which included the top two-thirds of participants. Descriptive statistics were conducted for maternal capital measures in the whole sample but also for low and high capital groups to ascertain the characteristics of the participants that belong to these categories. Univariate linear regression was conducted to assess the relationship between objective and subjective maternal capital at baseline (predictors) and infant growth, infant behavior, maternal behavior and attitude, and infant feeding variables (outcomes) at 6–8 weeks (unadjusted model). The regression model was then adjusted for intervention assignment. General linear model was used to assess the interaction between objective/subjective maternal capital (as a continuous variable and as low/high categories) and intervention assignment on infant, maternal and breastfeeding outcomes. *p*-values <0.05 were considered statistically significant.

**Table 1:** descriptive maternal capital measures in the whole sample and in low/high objective and subjective maternal capital groups.

Socioeconomic capital	Overall		Low objective capital		High objective capital		Low subjective capital		High subjective capital	
	*n*	%	*n*	%	*n*	%	*n*	%	*n*	%
**Income**										
< £20 K - 30 K	11	22	3	21	8	22	5	29	6	18
< £45 K - 75 K	12	24	2	14	10	28	4	24	8	24
> £75 K	27	54	9	64	18	50	8	47	19	58
**Ethnicity**										
White	32	60	7	41^c^	25	69^c^	12	71	20	56
Asian	8	15	5	29^c^	3	8^c^	2	12	6	17
Black	7	13	4	24^c^	3	8^c^	2	12	5	14
Other	6	11	1	6^c^	5	14^c^	1	6	5	14
*Social capital*	*n*	Mean (SD)	*n*	Mean (SD)	*n*	Mean (SD)	*n*	Mean (SD)	*n*	Mean (SD)
Helping attitude	52	81.9 (7.9)	14	82.6 (8.1)	33	81.9 (8.3)	16	82.3 (8.5)	31	82.1 (8.2)
*Somatic capital*	n	Mean (SD)	*n*	Mean (SD)	*n*	Mean (SD)	*n*	Mean (SD)	*n*	Mean (SD)
Pregnancy weight gain (kg)	64	11.2 (5.7)	16	10.0 (7.8)	34	12.1 (4.9)	17	12.4 (6.7)	33	11.0 (5.7)
Infant birth weight (kg)	72	2.6 (0.4)	17	2.4 (0.2)^**b**^	36	2.7 (0.4)^**b**^	17	2.7 (0.3)	36	2.6 (0.4)
Height (cm)	71	163.3 (6.9)	17	158.6 (5.3)^**b**^	36	164.9 (5.6)^**b**^	17	161.2 (6.0)	36	163.7 (6.2)
Weight at 2–3 weeks (kg)	60	68.9 (11.3)	16	69.4 (11.5)	31	67.9 (10.6)	15	68.0 (11.4)	32	68.6 (10.7)
*Reproductive scheduling*	n	Mean (SD)	*n*	Mean (SD)	n	Mean (SD)	*n*	Mean (SD)	*n*	Mean (SD)
Primiparous^a^	37	70	10	59	27	75	13	76	24	67
Gestational age	72	36.5 (1.0)	17	36.6 (1.1)	36	36.2 (1.0)	17	36.2 (1.0)	36	36.4 (1.1)
Maternal age	72	33.1 (4.9)	15	33.2 (4.7)	36	33.6 (4.4)	17	31.9 (4.8)^c^	34	34.2 (4.1)^c^
*Psychological capital*	n	Mean (SD)	*n*	Mean (SD)	*n*	Mean (SD)	*n*	Mean (SD)	*n*	Mean (SD)
Stress	64	15.0 (5.5)	17	14.6 (3.8)	36	14.4 (6.2)	17	20.3 (3.7)^**b**^	36	11.8 (3.8) ^**b**^
Depression	58	7.7 (4.5)	17	8.4 (4.4)	36	7.3 (4.6)	17	12.1 (4.0)^**b**^	36	5.5 (2.9) ^**b**^
*Cognitive capital*	*n*	Mean (SD)	*n*	Mean (SD)	*n*	Mean (SD)	*n*	Mean (SD)	*n*	Mean (SD)
Immediate recall	61	55.2 (6.3)	17	55.2 (7.1)	36	55.1 (6.3)	17	53.8 (7.2)	36	55.7 (6.1)
Verbal learning	61	5.9 (1.9)	17	3.9 (1.4)^**b**^	36	6.7 (1.4)^**b**^	17	5.9 (2.0)	36	5.8 (1.9)
Verbal forgetting	61	1.5 (1.7)	17	−1.5 (1.7)	36	−1.5 (1.7)	17	−1.1 (1.7)	36	−1.7 (1.7)
**Education^a^**										
A-level, GCSE, or less	10	19	6	35^c^	4	11^c^	4	24	6	17
Bachelor’s degree or equivalent	20	39	6	35	14	40	6	35	14	40
Postgraduate degree	22	42	5	29^c^	17	49^c^	7	41	15	43

^a^Frequency- Percentage, ^b^*p* < 0.001; ^c^*p* = 0.1.

## 3. Results

### 3.1. Descriptive

The different aspects of maternal capital (socioeconomic, social, somatic, reproductive, psychological and cognitive) are described in Table 1. The differences in individual maternal capital measures between those in the low (*n* = 17) vs. high/moderate maternal capital composite scores (*n* = 36) were compared as shown in Table 1. There were trends toward lower maternal age in the low subjective maternal capital group, and toward higher frequency of participants who identified as Black or Asian and/or who had degrees of a lower level than bachelor’s degree in the low objective maternal capital group.

### 3.2. Maternal capital and maternal investment

Higher objective maternal capital was significantly associated with lower non-responsiveness to infant cues at 6–8 weeks (OR = −0.05, 95% CI −0.09, −0.02; Table 2) while higher subjective maternal capital (lower stress and depression scores) was significantly associated with a higher breastfeeding frequency (OR = 2.3; 95% CI 0.8, 3.8).

**Table 2:** The relationship between subjective and objective maternal capital at 2–3 weeks with infant growth, infant behavior, maternal engagement with the infant, and infant feeding at 6–8 weeks, using simple linear regression.

Outcomes at 6–8 weeks	Predictors at 2–3 weeks post-delivery							
	Subjective capital (HV1)	*p*-value	Adjusted β^1^	Adjusted *p*-value^1^	Objective capital (HV1)	*p*-value	Adjusted β^1^	Adjusted *p*-value^1^
**Infant growth**								
Infant weight Z-score	0.04 [−0.22, 0.32]	0.74	0.04 [−0.22, 0.30]	0.77	**0.43 [0.21, 0.66]**	**0.000**	**0.39 [0.16, 0.62]**	**0.001**
Weight Z-score gain	−0.01 [−0.20, 0.18]	0.94	0.00 [−0.19, 0.18]	0.96	0.06 [−0.13, 0.25]	0.52	0.02 [−0.17, 0.20]	0.85
Infant length Z-score	0.12 [−0.22, 0.47]	0.49	0.13 [−0.22, 0.47]	0.47	**0.47 [0.19, 0.76]**	**0.002**	**0.46 [0.17, 0.75]**	**0.003**
Length Z-score gain	0.04 [−0.22, 0.30]	0.75	0.04 [−0.22, 0.31]	0.76	0.03 [−0.23, 0.29]	0.81	0.05 [−0.22, 0.32]	0.71
**Infant behavior**								
Crying/colic	−7.8 [−25.8, 10.8]	0.38	−3.2 [−21.9, 15.6]	0.73	**−17.5 [−33.2, −1.9]**	**0.03**	−14.4 [−31.2, 2.4]	0.09
Fussiness	−3.0 [−26.8, 20.7]	0.79	−5.2 [−31.4, 21.0]	0.69	−11.7 [−22.8, 10.4]	0.29	−14.9 [−38.9, 9.0]	0.21
Sleeping	1.7 [−51.7, 55.1]	0.95	3.9 [−52.5, 60.3]	0.89	7.0 [−44.5, 58.5]	0.78	15.4 [−30.9, 61.8]	0.50
General appetite	**0.30 [0.07, 0.54]**	**0.01**	**0.32 [0.09, 0.55]**	**0.009**	0.01 [−0.23, 0.26]	0.90	0.02 [−0.21, 0.26]	0.84
Satiety responsiveness	−0.03 [−0.19, 0.13]	0.72	−0.02 [−0.19, 0.14]	0.78	**−0.17 [−0.31, −0.02]**	**0.02**	**−0.16 [−0.31,-0.02]**	**0.03**
Food responsiveness	−0.04 [−0.27, 0.19]	0.75	−0.03 [−0.26, 0.20]	0.79	**−0.28 [−0.48, −0.08]**	**0.008**	**−0.27 [−0.48,-0.07]**	**0.009**
Slowness in eating	0.00 [−0.22, 0.22]	0.98	−0.01 [−0.24, 0.21]	0.90	−0.05 [−0.25, 0.16]	0.65	−0.07 [−0.28, 0.14]	0.51
Enjoyment of food	0.05 [−0.10, 0.20]	0.49	0.05 [−0.10, 0.20]	0.51	−0.01 [−0.14, 0.13]	0.94	−0.01 [−0.15, 0.13]	0.93
**Maternal engagement with infant**								
Attachment	0.34 [−1.15, 1.83]	0.64	0.31 [−1.2, 1.8]	0.68	−0.15 [−1.50, 1.17]	0.82	−0.19 [−1.5, 1.2]	0.78
Responsiveness to cues	0.07 [−0.06, 0.20]	0.28	0.07 [−0.07, 0.20]	0.31	0.09 [−0.02, 0.21]	0.11	0.09 [−0.03, 0.21]	0.13
Delayed responsiveness	0.03 [−0.23, 0.28]	0.83	0.04 [−0.22, 0.30]	0.74	**−0.22 [−0.45, 0.00]**	**0.047**	−0.22 [−0.44, 0.01]	0.06
Non-responsiveness	−0.02 [−0.06, 0.02]	0.29	−0.02 [−0.06, 0.02]	0.38	**−0.05 [−0.09, −0.02]**	**0.004**	**−0.05 [−0.08, −0.01]**	**0.007**
**Infant feeding**								
Exclusive breastfeeding^a^	1.28 [0.66, 2.47]	0.46	1.28 [0.66, 2.47]	0.46	1.09 [0.59, 2.00]	0.79	1.08 [0.58, 2.03]	0.80
Formula feeds	−0.9 [−6.4, 4.6]	0.74	−0.8 [−6.3, 4.7]	0.77	0.0 [−5.0, 5.0]	0.99	0.4 [−4.7, 5.6]	0.86
Expressed milk feeds	2.1 [−2.1, 6.3]	0.34	2.1 [−2.2, 6.3]	0.33	0.2 [−3.7,4.1]	0.92	0.5 [−3.5, 4.6]	0.79
Breastfeeding frequency	**2.3 [0.8, 3.8]**	**0.006**	**4.1 [2.0, 6.1]**	**0.001**	0.7 [−1.3, 2.7]	0.44	0.3 [−1.5, 2.1]	0.71
BM volume (24 h Recall)	105.8 [−7.0, 218.7]	0.06	106.9 [−4.0, 217.8]	0.06	13.5 [−114.4, 141.4]	0.82	−0.6 [−131.3, 131.2]	0.99
Fat g/100 mL	−0.07 [−0.43, 0.31]	0.72	−0.07 [−0.43, 0.31]	0.73	−0.07 [−0.42, 0.27]	0.67	−0.07 [−0.42, 0.29]	0.71
True Protein g/100 mL	−0.06 [−0.12, 0.01]	0.11	−0.06 [−0.12, 0.01]	0.10	0.02 [−0.04, 0.09]	0.68	0.02 [−0.04, 0.09]	0.50
Carbohydrates g/100 mL	0.00 [−0.11, 0.11]	0.99	0.00 [−0.11, 0.11]	0.99	−0.06 [−0.17, 0.04]	0.22	−0.08 [−0.18, 0.03]	0.14
Energy Kcal/100 mL	−0.96 [−4.31, 2.40]	0.56	−0.95 [−4.35, 2.44]	0.57	−0.90 [−4.06, 2.26]	0.57	−0.84 [−4.10, 2.42]	0.61

^1^The relationship was adjusted for the intervention assignment. Significant results are highlighted in bold, and trends are highlighted in blue.

### 3.3. Maternal capital and infant growth and behavior

Higher objective maternal capital at 2–3 weeks significantly predicted higher weight (OR = 0.43; 95% CI 0.21, 0.66) and length z-scores (OR = 0.47; 95% CI 0.19, 0.76) and lower infant food responsiveness (OR = −0.28; 95% CI −0.48, −0.08) and satiety responsiveness (OR = −0.17; 95% CI -0.31, −0.02) at 6–8 weeks. Higher objective maternal capital also predicted a lower duration of crying at 6–8 weeks (OR = −17.5; 95% CI −33.2, −1.9), whereas higher subjective maternal capital was significantly associated with greater infant appetite (OR = 0.30; 95% CI 0.07, 0.54).

### 3.4. Adjustment for relaxation intervention effects

Since this data was collected as part of a randomized controlled trial, the relationship between each predictor and outcome was adjusted for the intervention assignment. The data shows higher objective capital still predicted higher weight z-score, length z-score and maternal responsiveness to her infant’s cues and lower food and satiety responsiveness. There was still a trend toward lower infant crying duration with higher objective maternal capital, but it is likely that the small sample size meant that this analysis was underpowered. A higher subjective maternal capital predicted a higher breastfeeding frequency and greater infant appetite, even after controlling for the intervention. The trend toward higher breast milk volume and lower protein concentration remained after adjustment.

### 3.5. Does the relaxation intervention moderate the association between maternal capital and investment?

**Figure 3:**
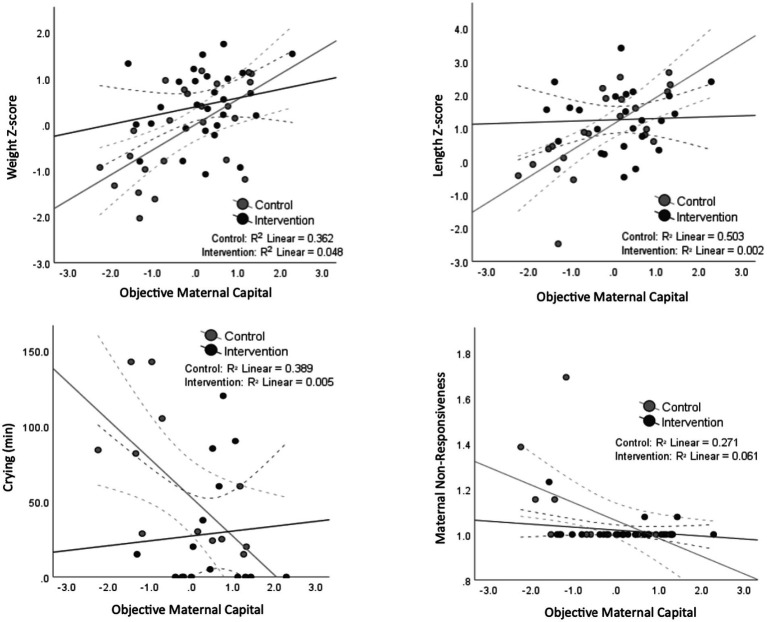
Scatter plots demonstrating the significant association (and trends) between maternal capital and infant, maternal and breastfeeding outcomes separated by the intervention assignment. The black dots and lines represent the intervention group, while the gray ones represent the control group. 95% confidence interval lines are shown as dashed lines. A significant interaction between the intervention assignment and maternal capital is present for length z-score, whereas a borderline significant interaction is shown for crying, weight and responsiveness.

**Figure 4:**
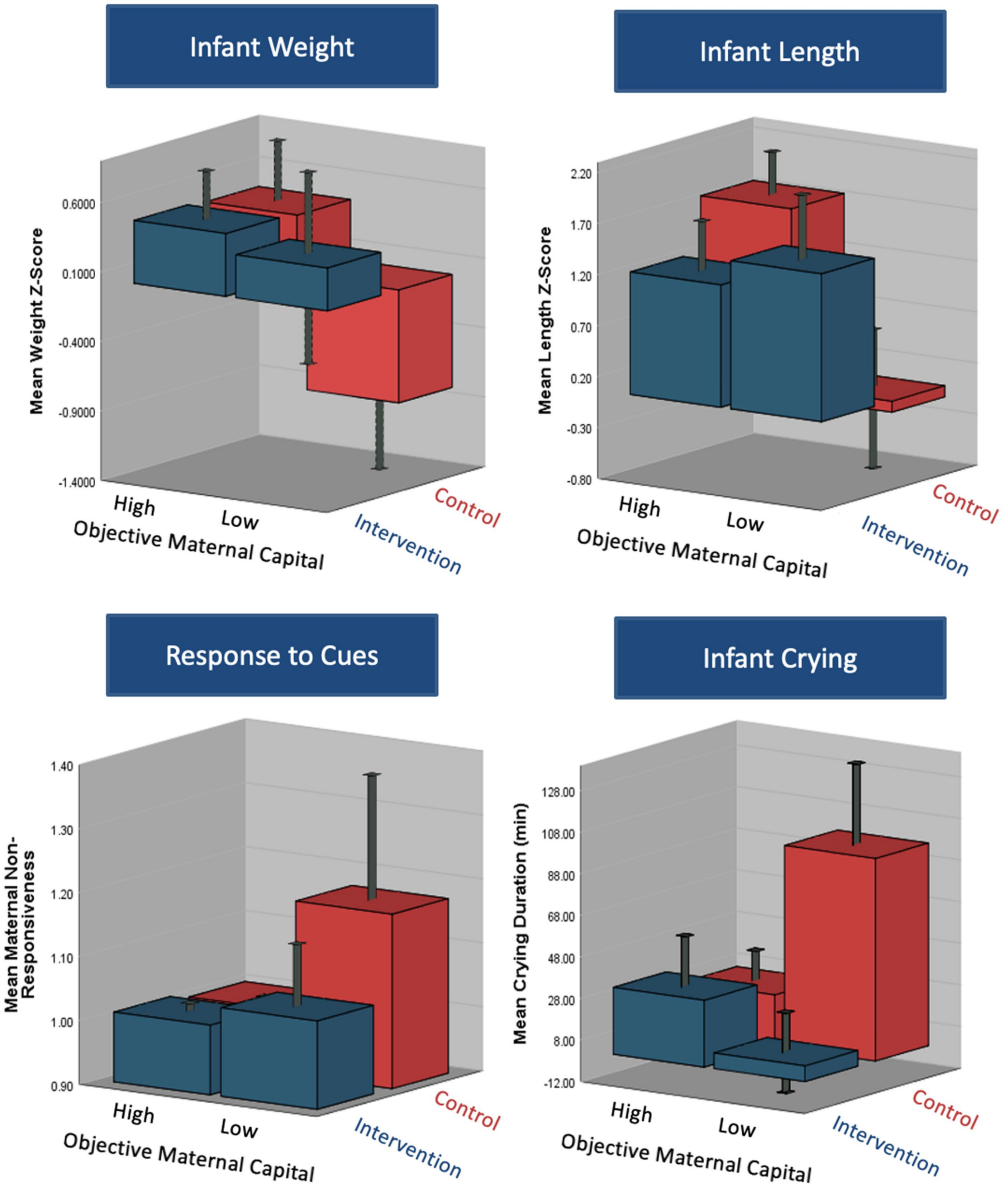
3D plots demonstrating the differences in infant, maternal and breastfeeding outcomes according to maternal capital group (high/moderate vs. low objective capital) and intervention group (intervention vs. control). The plots demonstrate a significant interaction between the capital and intervention assignment on infant weight, length and crying duration. There was a borderline significant interaction between the two on maternal responsiveness to infant cues.

The association between maternal capital and some outcomes varied according to whether the mothers received the relaxation intervention or not. Figure 3 suggests that the intervention was an ‘equalizer’ for the association between objective maternal capital and weight z-score, length z-score, infant crying, and maternal non-responsiveness, where those of lower maternal capital benefitted the most from the intervention. However, the degree and direction of correlation between maternal capital and the appetite traits and breastfeeding frequency did not change with the relaxation intervention. There was a significant interaction between the intervention assignment and objective maternal capital on length z-score (*p* = 0.006), and trends toward an interaction for weight z-score (*p* = 0.07), infant crying (*p* = 0.06) and maternal non-responsiveness (*p* = 0.06). This is further illustrated in Figure 4 where the effects of low maternal capital are mostly costly to infants whose mothers did not receive a relaxation intervention.

## 4. Discussion

Maternal capital represents the only environment that the offspring is exposed to during pregnancy, and largely the nutritional one they are exposed to postnatally if breastfeeding (17). Therefore, combining several maternal traits when studying the relationship between maternal capital and infant outcomes might be more meaningful than investigating individual traits. For instance, while we know that earlier menarche is usually associated with lower infant birth weight (mostly an indicator of lower parental investment) (59, 60), that is not always the case (61, 62). Psychosocial stress has been reported to be a moderator of the relationship between the two, where the presence of stressful life events as well as earlier menarche predicted lower birth weight, but earlier menarche alone did not (63). This suggests that the mother is able to provide a buffer for the offspring against some/few capital ‘insults.’ Therefore, it is important to account for how many insults on maternal capital are present to better predict differential investment in the offspring.

The results show that objective maternal capital at baseline (2–3 weeks post-delivery) was positively associated with weight and length z-scores at 6–8 weeks, but not with infant growth rate between these two time points. This suggests that mothers with higher energy available for investment in the offspring invested more during their intervention period, hence the larger size of their infant at 6–8 weeks. However, the lack of association between maternal capital and growth rate might mean that these infants allocate enough energy for growth without favoring it over other competing life functions such as metabolic homeostasis or immunity (we did not collect data on these markers to examine this). The results are in line with a previous study that showed that daughters of mothers of lower maternal capital (or with higher maternal capital penalties) were lighter and shorter at 1 year than those of higher capital mothers (39). However, by adulthood these daughters remained shorter but had higher BMI and markers of adiposity, indicating catch-up in weight rather than in linear growth. It is possible that long-term follow up data in our study may show similar accelerated growth trajectories post-infancy in the children of lower capital mothers.

Our study shows that maternal capital can also shape infant behavior. For instance, we found that higher objective maternal capital was associated with lower infant satiety responsiveness (i.e., higher drive to eat) and with somewhat shorter duration of crying. It is possible that maternal capital regulates infant appetite and behavior through bioactive components in breast milk acting as signals. During pregnancy, signals are transmitted from maternal blood to the fetus, and through breast milk postnatally. These signals reflect maternal condition and how the mother perceives her environment which in turn are strongly influenced by maternal development and early life experiences (17). Some of these signals are hormones associated with maternal psychological state and have the potential to regulate infant growth and development. For instance, studies, mostly in animals, have shown that maternal stress is associated with increased fearfulness, anxiety, crying and sleeping problems and attenuated growth in the offspring (64–70). We found that lower subjective maternal capital (i.e., higher stress and depression) was associated with lower infant appetite; however, we did not find any association with infant growth, crying, or sleeping. The correlations between maternal psychological state and infant appetite could be, at least partially, explained by some confounding factors. For example, gestational age could influence both psychological state (mothers of infants who are more premature are likely to be more stressed) and infant appetite (infants who are more premature are more likely to be feeding for shorter durations, more frequently). It is likely that maternal capital would serve as a buffer against short-term stressors and thus the infant is more likely to be influenced by the mother’s response to stressors rather than to short-term stress signals. That is why long-term markers of stress are needed to better predict infant response to stress exposure.

A potential pathway by which maternal capital can influence infant growth and behavior is through maternal behavioral and physiological investment. For example, we found that higher maternal capital predicted better maternal responsiveness to infant cues and higher breastfeeding frequency with a trend toward increased breast milk volume. From an evolutionary perspective, in uncertain environments characterized by higher stress levels reduced breastfeeding could be a method that has evolved to limit maternal investment (71). It could be explained from an energy trade-off point of view, where in distressed mothers, energy is being diverted from investment in the offspring toward mounting a stress response and preserving energy for the mother’s survival or future reproduction. This constraint can be in the form of reduced production of breast milk, or through reducing certain constituents of breast milk (macronutrients or hormones) that might be costly to supply. Distressed mothers may also have less available energy for nurturing behaviors such as holding, bonding, or responding promptly to cues.

Many of the maternal capital measures discussed above are already acknowledged to be important predictors of child health in conventional biomedical research. However, public health interventions aimed at improving breastfeeding or exclusive breastfeeding rates, and consequently infant outcomes, by targeting the mother are not always successful in achieving what they intended. This might be because from a medical perspective, milk is seen primarily as a source of nutrition, and lactation as a largely one-way process in which the mother provides whatever her infant needs (72). This perspective does not consider the social, cultural, historical and environmental basis of variability in lactation and the complex role the mother plays in regulating her offspring’s growth and behavior. Therefore, incorporating evolutionary concepts into the design of public health interventions could improve the success of these interventions. For instance, we previously combined medical and anthropological concepts into the design of three randomized controlled trials using relaxation therapy to reduce maternal stress and improve infant outcomes in breastfeeding women of term and preterm infants in the United Kingdom (current study (49)), Malaysia (73) and China (74). All three studies were successful in improving infant weight gain, with some finding differences in infant behavior, breast milk composition, and/or infant gut microbiome (Yu et al. unpublished data).

The results from this study show that the simple relaxation intervention could manipulate the association between maternal capital and outcomes. This could be interpreted in two ways: (i) capital influences responsiveness to the intervention, and/or (ii) the intervention directly influences capital by increasing investment in the offspring regardless. Based on our results, both interpretations are valid. We found that the intervention increased investment in the infant, but mainly in the low capital group, where it buffered against the consequences of low maternal capital, i.e., lower weight z-score, lower length z-score, prolonged infant crying and higher maternal non-responsiveness (although the relationship was significant for length z-score only). As such, infants whose mothers have low maternal capital and belonged in the control group were the most affected by maternal capital ‘penalties’. It is possible that the capacity of mothers with certain developmental stresses to invest in their offspring may become more severely impaired, if they experience psychosocial stress during lactation. On this basis, a stress reduction therapy during lactation might be especially beneficial. Additionally, parent-offspring theory explains that the mother and infant compete and negotiate over how much maternal resources will be invested in the offspring (3). How much the mother invests is the point at which maximum benefit is given to the infant without incurring maximal cost on the mother. It is possible that for mothers of low capital the surplus energy gained as a result of the intervention shifts this point of balance upwards to a point where the infant benefits more without incurring more cost for the mother. This might be different for mothers of high capital where the surplus of energy would not be invested in the infant, as the infant is already receiving maximal benefit, but is rather invested in the mother herself for maintenance, immunity and/or future reproduction.

Wells (17) differentiated between two different broad types of maternal capital: liquid and illiquid. The liquidity of capital refers to how quickly these resources could be gained and lost. For example, maternal height is a stable trait and thus is illiquid, whereas fat or vitamin stores could be accumulated or used through the reproductive career, and so are liquid capital. This theoretical approach could be extended to explore psychological aspects of maternal capital. For example, we could think of maternal temperament as a stable, relatively illiquid component of psychological capital, whereas short-term changes in mood, reflecting changing environments and circumstances, would indicate liquidity in psychological capacity and resilience. Temperament, reflecting genetic constitution and developmental experience in early life, might also shape fluctuations in short-term moods, just as linear growth reflecting the same influences may shape adult adiposity. Liquid psychological capital might also be assayed indirectly by assessing cortisol, a biomarker of activation of the stress response. Under the same adult environmental circumstances, some mothers may become stressed more easily than others, potentially due to illiquid traits such as temperament. Therefore, it is possible that the relaxation intervention might beneficially impact the liquid aspect of maternal psychological capital, by reducing maternal stress and anxiety.

Lasty, it is worth considering that participants in this study were mostly highly educated, most had high household incomes, all were above 20 years old (and most above 30 years), and most had only one child. Therefore, mothers in the low maternal capital group arguably did not truly have low capital, even though it was low relative to others in the study. It is unclear whether the intervention (or other similar interventions) would be more beneficial to mothers with truly low maternal capital and future studies should investigate this.

The main limitation of this analysis is the small sample size. We were unable to examine the differences between female and male infants who might exhibit different trade-off patterns. The small sample size also reduced the possibility of including more variables for Principal Component Analysis to represent maternal capital. The other limitation is that we did not collect data on other maternal capital traits pre-pregnancy such as age at menarche and maternal birth weight which could have provided more details on maternal capital and better predicted investment. Additionally, because this was a sample of mothers living in Greater London and intending to breastfeeding their late preterm or early term infant, the generalizability of the findings could be limited. However, one of the strengths of this study is that it demonstrated a novel analytical approach that could be applied to more representative samples of the general population. Another strength is that we used an experimental design to manipulate maternal capital. Principal Component Analysis was also used to identify composite scores of maternal capital which might provide a better indicator of maternal capital than assessing individual traits. Lastly, we collected a large number of maternal investment indicators such as maternal behavior, breast milk volume and composition, infant growth, and infant behavior and appetite.

In conclusion, we have used a novel approach to examine how mothers differentially invest in their offspring and regulate their growth and behavior according to maternal phenotype and environmental conditions. The findings show that higher maternal capital was associated with better infant growth and shorter infant crying duration, possibly mediated through more frequent breastfeeding and more prompt responsiveness to infant cues. The findings also suggest that a simple relaxation intervention could buffer against maternal capital insults, as the effects of low maternal capital appeared most detrimental to infants whose mothers did not receive the intervention. Overall, understanding and applying evolutionary concepts such as maternal capital to health-related studies could improve understanding of how the environment interacts with the mother, milk and infant. It could also help to better predict the outcome of interventional studies and achieve the desired results such as improved infant growth and promotion of breastfeeding.

## Data availability statement

The raw data supporting the conclusions of this article will be made available by the the corresponding author, upon reasonable request.

## Ethics statement

The studies involving humans were approved by Health Research Authority (United Kingdom). The studies were conducted in accordance with the local legislation and institutional requirements. The participants provided their written informed consent to participate in this study.

## Author contributions

SD: Conceptualization, Formal analysis, Funding acquisition, Investigation, Methodology, Writing – original draft, Writing – review & editing. MF: Conceptualization, Supervision, Writing – review & editing. JW: Conceptualization, Supervision, Writing – review & editing.
